# Supplementary material for: Modulation of cardiac cAMP signaling by AMPK and its adjustments in pressure overload-induced myocardial dysfunction in rat and mouse
Source: PLoS One. 2023 Sep 21;18(9):e0292015. doi: 10.1371/journal.pone.0292015 (PMC10513315; doi:10.1371/journal.pone.0292015)
Supplement: S1 Raw images — (PDF) [file pone.0292015.s001.pdf]

Figure 4

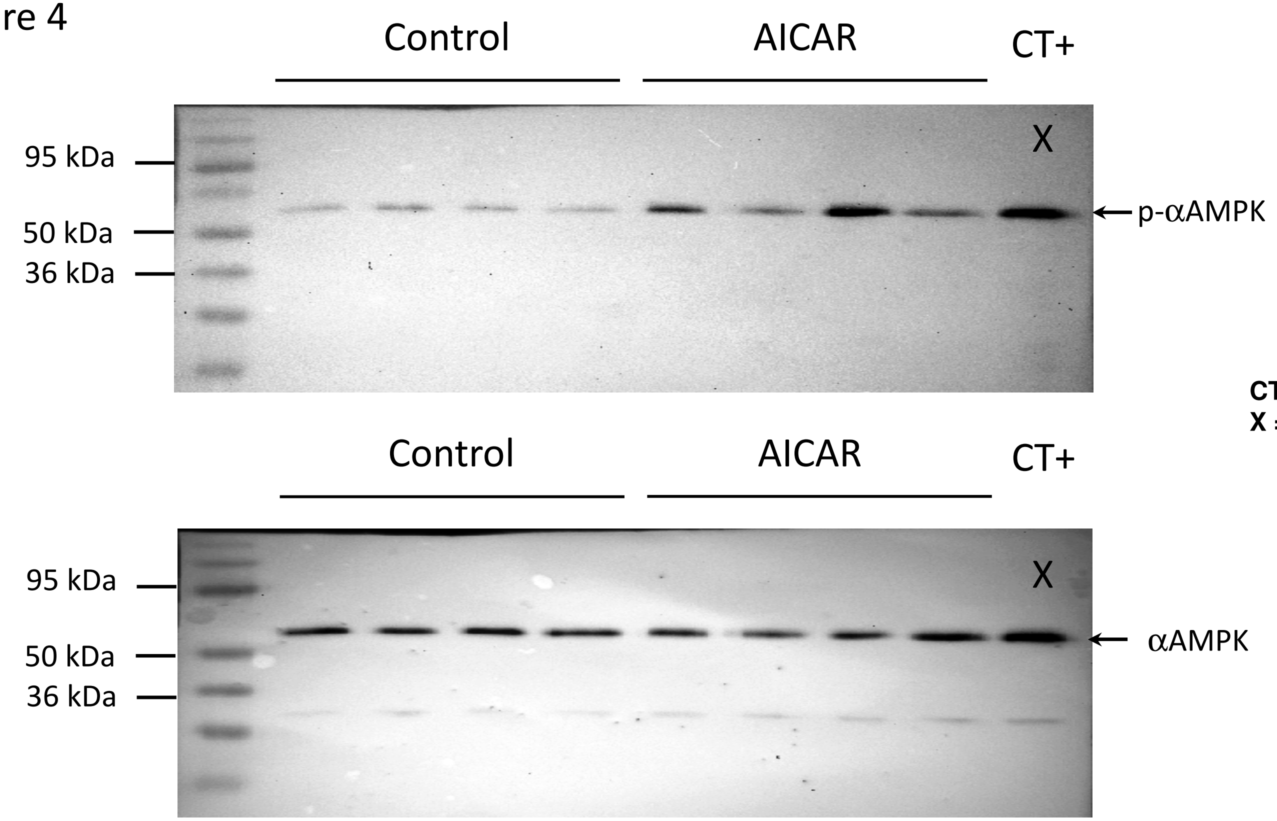

CT+ = positive control
X = these lanes are not displayed in the final figure

Figure 6

p-αAMPK

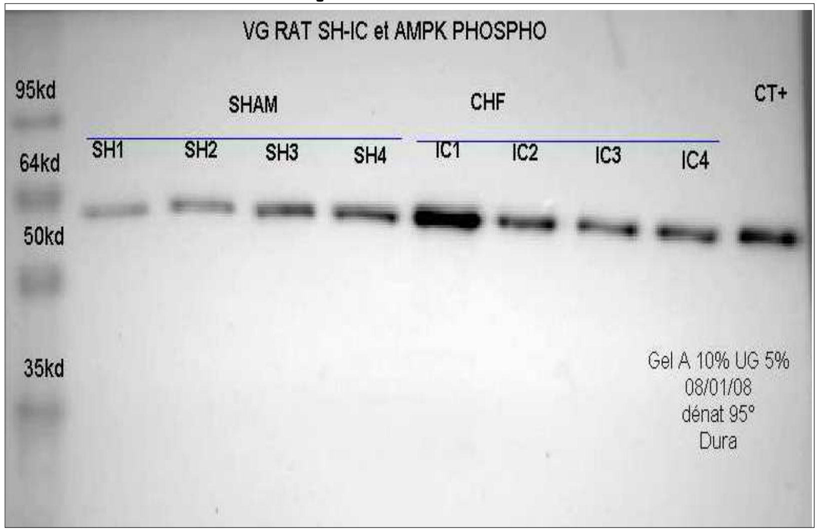

← p-αAMPK

αAMPK

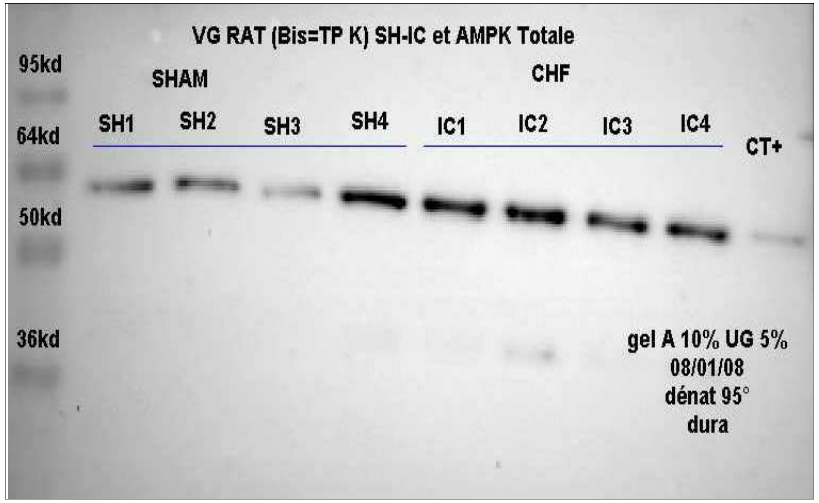

← αAMPK

GAPDH

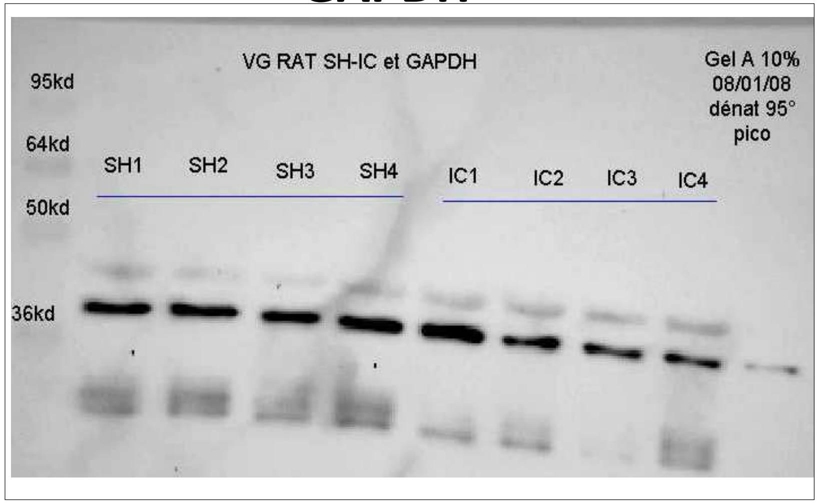

← GAPDH

CT+ = positive control
X = these lanes are not displayed in the final figure

Figure 6

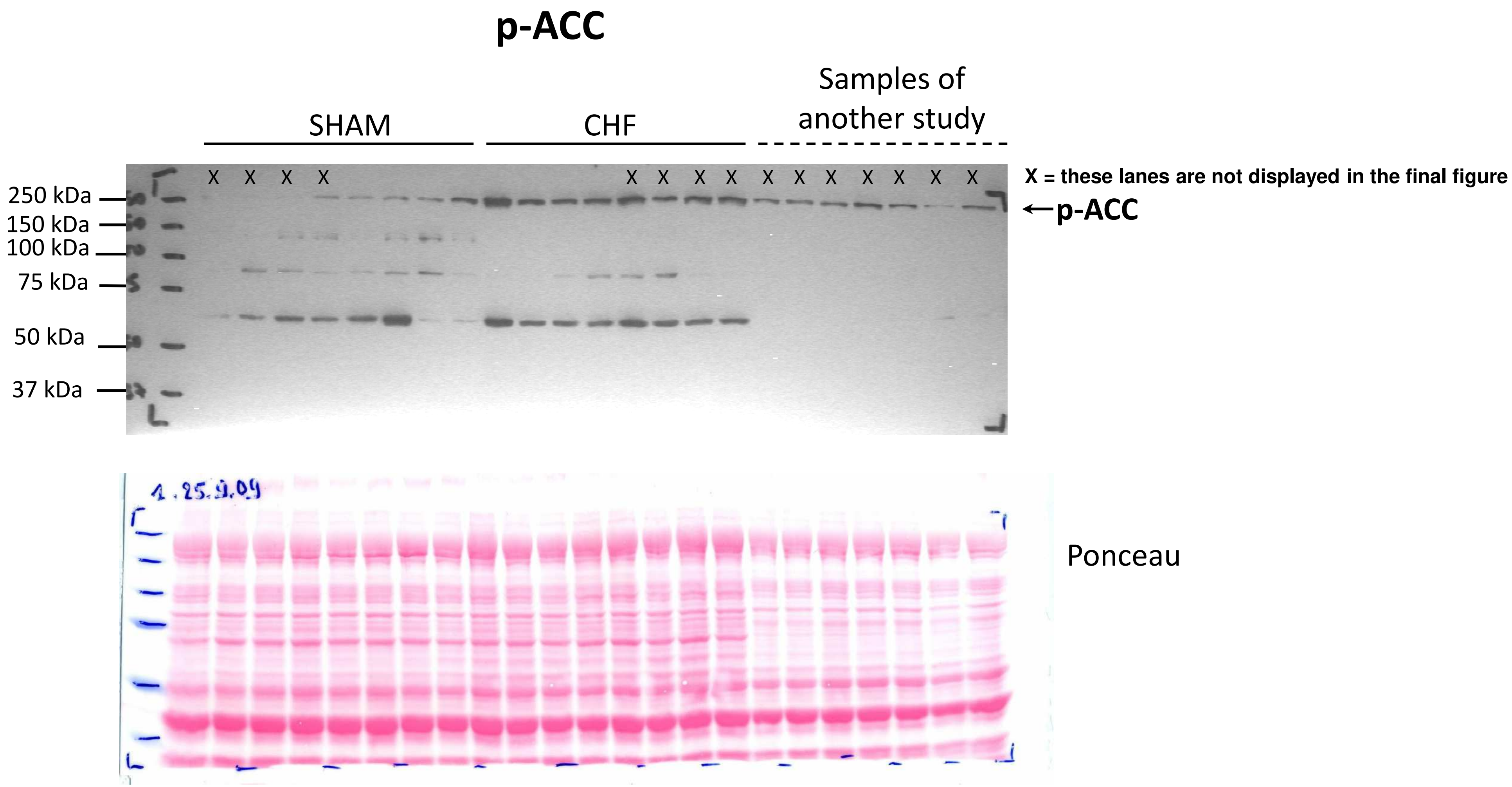

p-ACC
SHAM
CHF
Samples of another study
X = these lanes are not displayed in the final figure
250 kDa
150 kDa
100 kDa
75 kDa
50 kDa
37 kDa
p-ACC
Ponceau

Figure 6

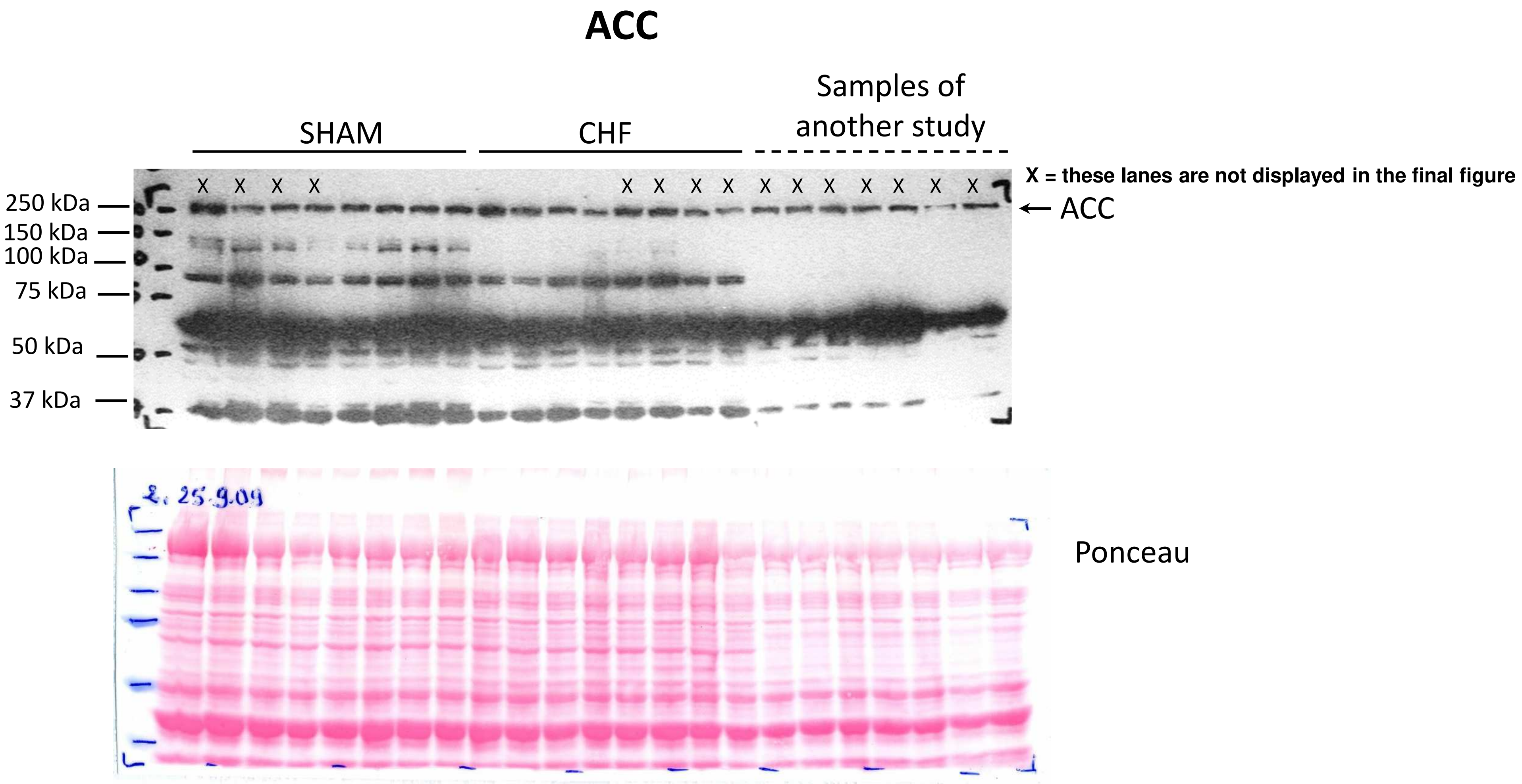
ACC
SHAM
CHF
Samples of another study
X = these lanes are not displayed in the final figure
X X X X X X X X X X X X X X X X X
250 kDa
150 kDa
100 kDa
75 kDa
50 kDa
37 kDa
← ACC
2. 25.9.09
Ponceau

Figure 7

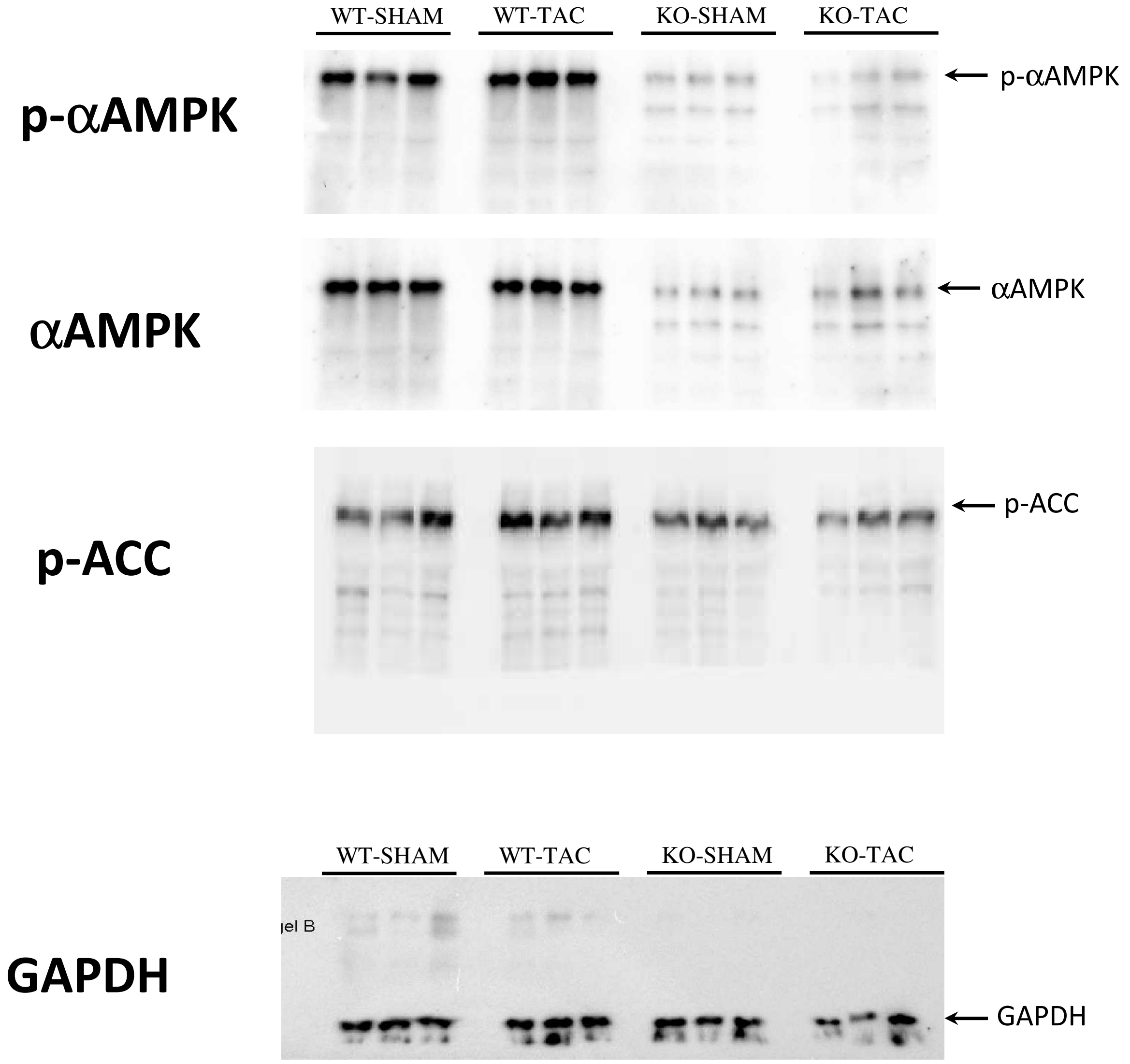
WT-SHAM
WT-TAC
KO-SHAM
KO-TAC
p-αAMPK
p-αAMPK
αAMPK
αAMPK
p-ACC
p-ACC
WT-SHAM
WT-TAC
KO-SHAM
KO-TAC
GAPDH
GAPDH
