# Supplementary material for: Modulation of cardiac cAMP signaling by AMPK and its adjustments in pressure overload-induced myocardial dysfunction in rat and mouse
Source: PLoS One. 2023 Sep 21;18(9):e0292015. doi: 10.1371/journal.pone.0292015 (PMC10513315; doi:10.1371/journal.pone.0292015)
Supplement: Attachment — Submitted filename: Response to reviewers_PONE-D-23-18218.docx [file pone.0292015.s002.docx]

Journal requirements:

When submitting your revision, we need you to address these additional requirements.

1. Please ensure that your manuscript meets PLOS ONE's style requirements, including those for file naming. The PLOS ONE style templates can be found at

https://journals.plos.org/plosone/s/file?id=wjVg/PLOSOne_formatting_sample_main_body.pdf and

<https://journals.plos.org/plosone/s/file?id=ba62/PLOSOne_formatting_sample_title_authors_affiliations.pdf>

**Response : Modifications have been made in accordance with the guidelines.**

2. Thank you for stating the following in the Acknowledgments Section of your manuscript:

“We are indebted to Dominique Fortin, Florence Lefebvre, Jocelyne Leclerc and M-F Messmer for helpful technical assistance. We thank Valérie Domergue and the animal core facility of UMR IPSIT for efficient handling and breeding of the animals. We are grateful to Pauline Robert for technical assistance in mice genotyping, and Patrick Lechêne for FRET experiments. The authors would also like to thank R. Fischmeister and Ana-Maria Gomez for continuous support and fruitful discussions.

              Our laboratory is a member of the Laboratory of LERMIT and is supported by grants from “Fondation pour la Recherche Médicale” (to A.G, #DPM20121125546), European Research Area Networkon Cardiovascular Diseases (to J.P, #ANR-19-ECVD-0007-01), Fondation de France (to A.G, 00086500). R V-C is emerit scientist at Centre National de la Recherche Scientifique (CNRS).”

We note that you have provided funding information that is not currently declared in your Funding Statement. However, funding information should not appear in the Acknowledgments section or other areas of your manuscript. We will only publish funding information present in the Funding Statement section of the online submission form.

Please remove any funding-related text from the manuscript and let us know how you would like to update your Funding Statement. Currently, your Funding Statement reads as follows:

“The author(s) received no specific funding for this work.”

Please include your amended statements within your cover letter; we will change the online submission form on your behalf."

**Response : The funding mentioned in this section is not directly related to the present study. We have removed the mentionned funding from the acknowledgements section to comply with the guidelines, and we do not wish it to appear in the "funding statement" sections.**

3. PLOS ONE now requires that authors provide the original uncropped and unadjusted images underlying all blot or gel results reported in a submission’s figures or Supporting Information files. This policy and the journal’s other requirements for blot/gel reporting and figure preparation are described in detail at https://journals.plos.org/plosone/s/figures#loc-blot-and-gel-reporting-requirements and https://journals.plos.org/plosone/s/figures#loc-preparing-figures-from-image-files. When you submit your revised manuscript, please ensure that your figures adhere fully to these guidelines and provide the original underlying images for all blot or gel data reported in your submission. See the following link for instructions on providing the original image data: https://journals.plos.org/plosone/s/figures#loc-original-images-for-blots-and-gels.

In your cover letter, please note whether your blot/gel image data are in Supporting Information or posted at a public data repository, provide the repository URL if relevant, and provide specific details as to which raw blot/gel images, if any, are not available. Email us at plosone@plos.org if you have any questions.

**Response : All original uncropped and unadjusted images underlying blots are now supplied in the file « S1-raw images » uploaded as a Supporting information file.**

4. We note that you have included the phrase “data not shown” in your manuscript. Unfortunately, this does not meet our data sharing requirements. PLOS does not permit references to inaccessible data. We require that authors provide all relevant data within the paper, Supporting Information files, or in an acceptable, public repository. Please add a citation to support this phrase or upload the data that corresponds with these findings to a stable repository (such as Figshare or Dryad) and provide and URLs, DOIs, or accession numbers that may be used to access these data. Or, if the data are not a core part of the research being presented in your study, we ask that you remove the phrase that refers to these data.

**Response : The sentence has been removed.**

5. Please review your reference list to ensure that it is complete and correct. If you have cited papers that have been retracted, please include the rationale for doing so in the manuscript text, or remove these references and replace them with relevant current references. Any changes to the reference list should be mentioned in the rebuttal letter that accompanies your revised manuscript. If you need to cite a retracted article, indicate the article’s retracted status in the References list and also include a citation and full reference for the retraction notice.

**Response : The references cited in this manuscript have all been checked and comply with PLOS ONE journal requirements.**


Reviewers' comments:

Reviewer's Responses to Questions

**Comments to the Author**

1. Is the manuscript technically sound, and do the data support the conclusions?

The manuscript must describe a technically sound piece of scientific research with data that supports the conclusions. Experiments must have been conducted rigorously, with appropriate controls, replication, and sample sizes. The conclusions must be drawn appropriately based on the data presented.

Reviewer #1: Yes

2. Has the statistical analysis been performed appropriately and rigorously?

Reviewer #1: Yes

3. Have the authors made all data underlying the findings in their manuscript fully available?

The [PLOS Data policy](http://www.plosone.org/static/policies.action#sharing) requires authors to make all data underlying the findings described in their manuscript fully available without restriction, with rare exception (please refer to the Data Availability Statement in the manuscript PDF file). The data should be provided as part of the manuscript or its supporting information, or deposited to a public repository. For example, in addition to summary statistics, the data points behind means, medians and variance measures should be available. If there are restrictions on publicly sharing data—e.g. participant privacy or use of data from a third party—those must be specified.

Reviewer #1: Yes

4. Is the manuscript presented in an intelligible fashion and written in standard English?

PLOS ONE does not copyedit accepted manuscripts, so the language in submitted articles must be clear, correct, and unambiguous. Any typographical or grammatical errors should be corrected at revision, so please note any specific errors here.

Reviewer #1: Yes

5. Review Comments to the Author

Please use the space provided to explain your answers to the questions above. You may also include additional comments for the author, including concerns about dual publication, research ethics, or publication ethics. (Please upload your review as an attachment if it exceeds 20,000 characters)

Reviewer #1: In this article, the authors worked with the hypothesis of a possible crosstalking between AC5 and AMPK in hearts of WT and KO mice for AMPKα2-/-. The work was very well executed, it is very well written and presents very robust data to demonstrate the initial hypothesis. The authors worked very well on the intracellular signaling pathways involved in this crosstalking, using various resources to reach their conclusions.

Minor points to consider.
The Ca2+ current explored by the authors as a way to demonstrate the importance of the action of AMPK on AC5 showed no difference between the WT and KO animals initially tested (Fig.2C). The difference appeared only in KO animals when IBMX (PDE inhibitor) was used (Fig.2D). Shouldn't the increased Ca2+ current in the KO animals have appeared in both moments? How in KO animals would PDE be masking this situation? The authors should explore this situation further since this finding was not discussed.

**Response : We thank the reviewer for this relevant comment. Actually, there is no effect on basal current because, unlike in atrial cells, basal cyclase activity is quite low in adult ventricular cells and is totally counterbalanced by PDE activity. Although cyclase activity is higher in KOs, it is unlikely to be sufficient to surpass PDE activity. It is therefore logical to see an increase in cAMP levels measured by FRET, and consequently increased calcium channel activity, only when PDEs are inhibited. This more detailed explanation has been included in the text p. 18**

6. PLOS authors have the option to publish the peer review history of their article ([what does this mean?](https://journals.plos.org/plosone/s/editorial-and-peer-review-process#loc-peer-review-history)). If published, this will include your full peer review and any attached files.


If you choose “no”, your identity will remain anonymous but your review may still be made public.


**Do you want your identity to be public for this peer review?** For information about this choice, including consent withdrawal, please see our [Privacy Policy](https://www.plos.org/privacy-policy).

Reviewer #1: No

[NOTE: If reviewer comments were submitted as an attachment file, they will be attached to this email and accessible via the submission site. Please log into your account, locate the manuscript record, and check for the action link "View Attachments". If this link does not appear, there are no attachment files.]

While revising your submission, please upload your figure files to the Preflight Analysis and Conversion Engine (PACE) digital diagnostic tool, <https://pacev2.apexcovantage.com/>. PACE helps ensure that figures meet PLOS requirements. To use PACE, you must first register as a user. Registration is free. Then, login and navigate to the UPLOAD tab, where you will find detailed instructions on how to use the tool. If you encounter any issues or have any questions when using PACE, please email PLOS at [figures@plos.org](mailto:figures@plos.org). Please note that Supporting Information files do not need this step.

**Response : All figure files have been checked using PACE digital diagnostic tool. The figure files of this submitted version of the article are the PACE generated files downloaded after processing by PACE.**
